# Community perceptions of malaria and vaccines in the South Coast and Busia regions of Kenya

**DOI:** 10.1186/1475-2875-10-147

**Published:** 2011-05-30

**Authors:** David I Ojakaa, Peter Ofware, Yvonne W Machira, Emmanuel Yamo, Yvette Collymore, Antoinette Ba-Nguz, Preeti Vansadia, Allison Bingham

**Affiliations:** 1 African Medical and Research Foundation, Wilson Airport, Langata Road, PO Box 30125, Nairobi, Kenya; 2 Previous address: African Medical and Research Foundation, Wilson Airport, Langata Road, PO Box 30125, Nairobi, Kenya; 3 PATH Malaria Vaccine Initiative, 455 Massachusetts Avenue NW, Washington, DC 20001-2621, USA; 4 PATH Malaria Vaccine Initiative, ACS Plaza, 4th floor, Lenana and Galana Road, PO Box 76634, Nairobi 00508, Kenya; 5 PATH, 2201 Westlake Avenue, Suite 200, Seattle, WA 98121, USA

## Abstract

**Background:**

Malaria is a leading cause of morbidity and mortality in children younger than 5 years in Kenya. Within the context of planning for a vaccine to be used alongside existing malaria control methods, this study explores sociocultural and health communications issues among individuals who are responsible for or influence decisions on childhood vaccination at the community level.

**Methods:**

This qualitative study was conducted in two malaria-endemic regions of Kenya--South Coast and Busia. Participant selection was purposive and criterion based. A total of 20 focus group discussions, 22 in-depth interviews, and 18 exit interviews were conducted.

**Results:**

Participants understand that malaria is a serious problem that no single tool can defeat. Communities would welcome a malaria vaccine, although they would have questions and concerns about the intervention. While support for local child immunization programs exists, limited understanding about vaccines and what they do is evident among younger and older people, particularly men. Even as health care providers are frustrated when parents do not have their children vaccinated, some parents have concerns about access to and the quality of vaccination services. Some women, including older mothers and those less economically privileged, see themselves as the focus of health workers' negative comments associated with either their parenting choices or their children's appearance. In general, parents and caregivers weigh several factors--such as personal opportunity costs, resource constraints, and perceived benefits--when deciding whether or not to have their children vaccinated, and the decision often is influenced by a network of people, including community leaders and health workers.

**Conclusions:**

The study raises issues that should inform a communications strategy and guide policy decisions within Kenya on eventual malaria vaccine introduction. Unlike the current practice, where health education on child welfare and immunization focuses on women, the communications strategy should equally target men and women in ways that are appropriate for each gender. It should involve influential community members and provide needed information and reassurances about immunization. Efforts also should be made to address concerns about the quality of immunization services--including health workers' interpersonal communication skills.

## Background

Despite progress in fighting malaria worldwide, the parasitic disease kills close to 800,000 people annually [1]. In Kenya, an estimated 27 million people (about 70 percent of the population) are at risk of infection, and roughly 34,000 young children die of malaria-related causes annually [2]. Current interventions in Kenya include the use of long-lasting insecticide-treated nets, indoor residual spraying with insecticides, intermittent preventive therapy with sulphadoxine-pyrimethamine for expectant mothers, and artemisinin-based combination therapies for malaria case management [2].

Recent advances in malaria vaccine development have heightened the possibility that, if proven effective, a new anti-malaria tool could be deployed for use alongside existing interventions [3]. A vaccine candidate targeting the most life-threatening malaria parasite, *Plasmodium falciparum*, is moving through a Phase 3 efficacy trial in Kenya and six other African countries--Burkina Faso, Gabon, Ghana, Malawi, Mozambique, and Tanzania [3]. Data from previous studies indicate that this vaccine candidate--GlaxoSmithKline Biologicals' RTS, S--may cut episodes of clinical malaria in young children by about half [4]. If the efficacy trial confirms earlier findings, the World Health Organization (WHO) has indicated that a policy recommendation for RTS, S is possible as early as 2015, paving the way for implementation in countries through their expanded programs on immunization. Final results from the trial are expected in late 2014.

But experience has shown that licensing a new health intervention hardly guarantees its timely use. In developing countries, it has taken up to two decades for new vaccines to become available to communities after they are licensed [5]. Similarly, while the malaria vaccine community's 2015 mark for a first-generation product [6] may be within reach, the actual introduction and use of a new vaccine could be held up for years because of a variety of factors. For example, the absence of critical data could slow down the process that policymakers must undertake to determine whether or not to introduce a particular intervention into their health systems [7]. In addition, misinformation within communities or poorly handled information by decision-makers could result in an outright lack of support for an intervention by local communities even after it has been introduced [8].

The formative study described in this paper is meant to address these communications needs. Information gathered is meant to add to the range of well-timed data required by policymakers to reach a decision on whether or not to add a malaria vaccine to their existing methods [7]. The information is also meant to inform the design of a communications strategy, including ways to engage communities well before a malaria vaccine becomes available. This paper presents the results of the study in Kenya within the context of informed decision-making related to future malaria vaccines and with an eye to the elements that should form the basis of a communications strategy, including those factors that potentially motivate or represent constraints to vaccination.

The impetus for this study came from several sources. The wider malaria vaccine community called for this information to help African decision-makers better understand how and whether they should introduce a malaria vaccine for use with other malaria interventions [6]. Responding to this call, in 2006, the PATH Malaria Vaccine Initiative (MVI), WHO, and national immunization and malaria control programs in several African countries outlined the data required to assess the need for such a vaccine [7]. In addition, WHO's framework for new vaccine introduction calls this type of research a key step toward vaccine introduction, as it provides a solid evidence base for, among other things, designing an informed communications strategy [9]. Other experts have also concluded that it is essential to understand the relevant sociocultural context for a new health intervention, such as a vaccine, to be successful [10].

The relevant literature on immunization programs and services in sub-Saharan Africa highlights the important role of communications. Some research shows that while communities are generally knowledgeable about vaccine-preventable diseases, mothers may lack key vaccination schedule information, such as how many vaccinations their children should receive or by what age they should have completed the course of vaccines [11]. A communications strategy could also take into account any beliefs about disease severity that may influence whether a child is vaccinated.

Other research has pointed to the importance of communications on issues around access to services, such as the distance that some families must travel for vaccination at primary health care facilities [12] and interactions between vaccination providers and users. A distrustful climate between communities and immunization programs can contribute to growing pools of non-immunized and partially immunized children [13-15]. Recent research also points to the key role that trusted sources or opinion leaders can play in fostering acceptance for vaccination [16]. In this regard, health care providers are themselves seen as important influencers of parents and other caregivers [15].

With all this in mind, the African Medical and Research Foundation (AMREF) in Kenya worked with MVI to explore sociocultural and health communications issues among individuals at the community level--both those responsible for decisions about vaccine use and those likely to influence such decisions. The study's objectives were also closely tied to the need to identify and describe target audiences and to highlight the relevant beliefs, values, attitudes, knowledge, and behaviors of these audiences prior to the introduction of an intervention [17]. The study therefore had the following specific objectives:

• To determine the perceptions, beliefs, practices, and information gaps related to malaria and vaccines among caregivers, service providers, and others at the community level.

• To determine household decision-making and treatment-seeking patterns related to malaria and vaccination.

• To identify key audiences and communications and information channels at the community level.

Given these objectives, it is nevertheless important to emphasize that this study should be seen as formative in nature. It is meant to inform other studies on perceptions of vaccination in relation to possible malaria vaccine introduction.

## Methods

### Site selection

The formative study was conducted in two regions of Kenya. Sites were selected based on a variety of factors, with the primary and secondary factors being malaria endemicity and local community and stakeholder support for conducting the study. High endemicity was sought to obtain data rich in experiences with malaria. Thereafter, variations in data were sought based on criteria such as ability to sample rural and urban populations, diversity in ethnic groups, and variation in religion.

The two study regions selected were the larger Busia region in Western Province and South Coast in Coast Province. Busia, situated in the Uganda-Kenya border region near Lake Victoria, is considered a highly endemic malaria region with year-round transmission [18]. Within the Busia region, study participants resided in the following areas: Busia Township, Bunyala, Samia, Butula, and Nambale. This predominantly Christian region is primarily home to the Luhya tribe. Dominant languages spoken by study participants were Luhya and Kiswahili.

The South Coast region is considered highly endemic with perennial malaria transmission; however, more recent data indicate that malaria is declining in the region [19]. The rural and urban areas sampled included Kwale, Kinango, Msambweni, and Mombasa. The South Coast region has greater Arab influence, is of Swahili culture, and has a higher proportion of Muslims than other parts of the country. Languages spoken by study participants were Digo, a dialect of the Mijikenda language, and Kiswahili.

### Participant selection and data collection

Participant selection was largely purposive and criterion based. Criteria were based on a literature review and an ecological conceptual framework, commonly used in health planning formative studies [20]. This framework was successfully adapted for use by PATH in other new child vaccine planning studies [21]. The framework recognizes levels or categories of people who influence whether a child is immunized. These levels constitute important target audiences for developing a health communications strategy aimed at engaging communities in new vaccine introduction activities. They include:

1. The individual level: Parents and other caregivers of children.

2. The interpersonal level: Secondary influencers such as teachers, health workers, and the media and other communicators.

3. The community level: Community leaders, local administrators, and local government officials.

4. The institutional level: Health care personnel and administrators.

Seven research assistants (four in the Coast region and three in the Busia region) were recruited for data collection. All seven (three men and four women) had degrees in related social sciences and had prior experience with qualitative interviewing. They were native speakers of the relevant local languages and speakers of the second national language, Kiswahili. Training of the research assistants and pre-testing of tools were carried out in October 2009. The training, which took place in the coastal city of Mombasa, covered an introduction to MVI's Community Perceptions Study and a re-orientation on such qualitative research approaches as data collection, obtaining informed consent, and analysis. Study tools were also reviewed, translated, and back-translated. Thereafter, pilot-testing was completed in the Coast region, and data collection activities began in November 2009. All activities were monitored by senior research staff.

A total of 20 focus group discussions (FGDs) were held, with 234 participants; 22 key informant interviews were conducted; and 18 exit interviews were completed in maternal and child health clinics (Table 1). FGDs were held with similarly situated respondents (e.g., mothers between 18 and 24 years of age) in an effort to solicit more candid responses. In addition, data were collected on vaccine delivery, immunizations, and administrative services from the health facilities visited.

**Table 1:** Sampling framework and final sample size

Busia region	South Coast region/Mombasa district	Number of events
*Focus group discussions*		

Mothers 50+ years old	Mothers 50+ years old	
Fathers 50+ years old	Fathers 50+ years old	
Mothers 25-49 years old (2 groups)	Mothers 25-49 years old (2 groups)	
Fathers 25-49 years old	Fathers 25-49 years old	
Mothers 18-24 years old	Mothers 18-24 years old	
Fathers 18-24 years old	Fathers 18-24 years old	
Mothers 18-49 years old	Mothers 18-49 years old	
Fathers 18-49 years old	Fathers 18-49 years old	
Community health workers	Community health workers	20

*Key informant interviews*		

Woman leader, youth leader, assistant chief, pastor, teacher, shopkeeper, traditional birth attendant, civil society organization representative, district public health officer, district nutritionist	Woman leader, youth leader, chief, pastor, imam, traditional birth attendant, civil society organization representative, teacher, local pharmacist, district public health officer, district medical officer, district public health nurse	22

*Clinic exit interviews*		

Mothers in an antenatal clinic (3)	Mothers in an antenatal clinic (3)	
Mothers in a child welfare center (3)	Mothers in a child welfare center (3)	
Mothers with febrile children (3)	Mothers with febrile children (3)	18

Total		60 events

FGDs and in-depth interviews were both noted and recorded, then later transcribed and translated from the local languages--Luhya, Digo, and Kiswahili--to English. Researchers carried out thematic content analysis using a codebook process on the translated English transcripts. To ensure maximum data validity and verification of findings at different levels, two iterations of analysis were conducted. Researchers first reviewed the transcripts for key themes using an iterative discursive process and developed a master codebook. At the second level, the researchers worked with an analyst versed in Atlas. ti software to code the data electronically and then generate reports to further explore thematic relationships and variations in the data by site, age, category of focus group participants, and type of interview.

### Ethics approval

The study was reviewed in Kenya by the AMREF Ethics and Scientific Review Committee and in the United States by the PATH Research Ethics Committee.

## Results

Results are organized in three general thematic areas: perceptions of and experiences with malaria; perceptions of and experiences with child immunization; and considerations for a future malaria vaccine (Table 2). Since the analysis included a comparative examination of findings by region, gender, and study group, observable differences are highlighted where they exist.

**Table 2:** Themes explored in the study

Theme	Thematic areas explored
1. Perceptions and experiences with malaria	• Perceptions of women and men around malaria. • Perceived causes of malaria. • Community experiences with malaria control efforts.

2. Perceptions and experiences with child immunization	• Community experiences with vaccination programs and the health system. • Community perceptions of factors that influence immunization coverage and acceptance of a new vaccine. • Decision-making around child vaccination.

3. Considerations for a future malaria vaccine	• Reactions to the prospect of a new vaccine. • Expectations for a new vaccine. • Concerns about a possible new vaccine. • The concept of efficacy.

### Sociodemographic characteristics of study participants

Participants resided mainly in rural areas, though some lived in the urban slum areas of Mombasa. Among parents and community representatives, small-scale farming and small business were common occupations. Those with formal employment included pharmacists, health care providers, and government officials. Income variations were notable by age group and region. For participants older than 50, remittances by children were cited as a main source of income. And while cross-border, small business was common in Busia, income stemming from the tourist industry in the Coast was noted.

### Perceptions of and experiences with malaria

#### Malaria is a serious health concern

Overall, malaria appeared to be well known to the community. Study participants shared their experiences with the disease from the perspective of parents who had lost children, caregivers for other family members, observers of patients, and patients themselves. Participants in all groups noted that malaria was a serious health problem that could lead to the deaths of adults and children, cause weight loss, and contribute to poor school performance. The consensus across the groups that malaria had a negative impact on economic and family stability is reflected in an older father's comment:

*Malaria has really affected my family, and as we are talking, I have two children who are suffering from malaria. I brought them to the hospital and they were treated for malaria; I went back home with them and they improved. After a while, they got malaria again and I came back to the hospital and they were treated, but again they got malaria once more after getting back home. Now, my earnings are affected because coming to the hospital is money and it becomes difficult to bring them back to the hospital since money is a problem*. (Participant, FGD fathers 50+, Coast region)

Participants recognized a similar cluster of symptoms of malaria across settings. In addition, fever may sometimes be equated with malaria and consequently, other illnesses that manifest fever as a symptom may also be described as malaria. The most commonly mentioned symptoms included headaches, body aches, fever, convulsions, vomiting, diarrhea, dehydration, loss of appetite, weight loss, low blood levels, red eyes, coughing, sneezing, fast breathing, restlessness, and depression. Some participants also referenced temporary madness (during severe episodes of malaria).

#### Who is at risk for malaria?

Although everyone was said to be at risk for malaria, focus group discussants generally recognized that groups who were particularly vulnerable to the disease included poor people and children--those between 9 months and 5 years of age, those younger than 15 years, and newborns. The antenatal and postnatal periods were identified as risk periods for the mother.

#### What causes malaria?

Most study participants understood that malaria is transmitted by mosquitoes. At the same time, parents in FGDs identified a number of factors as traditionally accepted causes of malaria, especially among children. For some, simply overeating or eating certain plant foods, such as raw mangoes, groundnuts, and young sugar cane, was reported as associated with getting malaria. Many parents also cited cold-water baths and weather-related causes, including exposure to dew and rain and spending the night in cold conditions (as is usually the case during funerals). In the Busia region, participants from the flood-prone district of Bunyala attributed high malaria incidence to flood waters.

The "forces of evil" as a cause of malaria emerged as an important perception in both regions. Mainly articulated in FGDs involving older participants, this perception was also highlighted by community health workers, who have a keen understanding of their communities. This factor was also more frequently cited in the Coast region, compared with Busia. In this regard, the *nyuni *(a bird) was often reported to be a common cause of malaria, as were evil spirits, known in the coastal Digo language as *tsagwa*. Going against cultural prohibitions--such as a woman having sexual relations soon after birth--was also seen to cause malaria. Professionals such as teachers, health care providers, and pharmacists in both South Coast and Busia tended to cite the scientific causes of the disease (e.g., bites by infectious mosquitoes), rather than these traditional beliefs.

#### Preventing malaria-like illnesses

Parents in the two regions appeared to be engaged in similar practices to prevent malaria in their children. Sleeping under mosquito nets was reported to be one of the best malaria control methods. However, parents noted that nets may not always be affordable or available and that using them may be inconvenient. Other anti-malaria measures for parents in both the Busia and Coast regions included keeping the house and surroundings clean, indoor and outdoor spraying with insecticides, the use of mosquito repellent jelly and mosquito coils, drainage of stagnant water, and the pouring of paraffin oil on water to kill larvae. They also reported keeping doors and windows closed during the evenings and the indoor burning of cow dung, *ubani *(incense), and the leaves of the *obengele*, *obwali*, or *mvumbani *trees to repel mosquitoes.

#### Treating malaria-like illnesses

While study participants talked about taking sick children to clinics, the first steps in managing malaria-like illnesses often appeared to be home based. First-line malaria care therapies for some involved the purchase of non-prescribed painkillers such as Panadol and Calpol. The use of herbs and traditional healers was also commonly mentioned across groups and settings, with participants saying that families often took children to traditional healers when home-based treatment was not effective. Some parents reported that children may be bathed in cold water to bring down temperatures, covered with blankets, or given large quantities of boiled water to drink. In one FGD in the Coast region, parents explained that a mother may have the sick child lie flat and urinate on him to cool his temperature.

### Perceptions of and experiences with child immunization

Perceptions and experiences with regard to having children vaccinated were explored in several ways during the FGDs and interviews. Thematic areas included decision-making within households related to child health and immunization, knowledge and previous experiences with vaccines and immunization efforts in Kenya, and perceived benefits of and constraints to child immunization.

#### Child health decision-making in households

Results indicated that decision-making related to child vaccination varies. In the Coast region, parents said the decision usually fell to the father or to another adult male household member. In Busia, parents generally agreed that mothers were the ones who usually decided whether or not to vaccinate a child:

*It is the mother who mostly decides, because when they go to clinic, they are taught about various types of vaccinations and their sequencing, including dates*. (Participant, FGD older fathers, Bunyala, Busia)

Some study participants had more nuanced answers on the question of who might decide whether or not to have a child vaccinated. They noted that in some instances, the family as a whole, including the extended family, may be involved in such a decision. In many situations, who makes the decision may depend on who is the main income earner, how busy the mother is at home, and who can provide transportation for the mother and child. Some participants noted that the family may also be influenced by a broader network of people that includes neighbors, community leaders, and health workers (see Table 3).

**Table 3:** Those likely to motivate others to access vaccination services

Primary influencers	Secondary influencers
• Community health workers	• Mothers-in-law
• Health care providers (doctors, nurses)	• Grandmothers
• Neighbors	• Councils of elders
• Village elders	• Traditional healers
• Mass media	• School-going children
• Local or provincial government administrations (including chiefs and assistant chiefs)	• Mother-to-mother support groups•
• Ministry of Medical Services	• Representatives of non-registered immigrants and nomadic communities
• Ministry of Public Health and Sanitation	• The elite within society (professionals/professional organizations)

#### Knowledge of local immunization programs

In general, parents were aware of local immunization programs and could name different diseases for which children are vaccinated. Pharmacists were more knowledgeable than parents about these programs. Older and younger men tended to demonstrate less knowledge about immunization programs than their male and female counterparts in the 25-49-year-old group.

#### Benefits of vaccination

Focus group participants and in-depth interviewees in both research settings generally reacted positively to the idea of having children vaccinated. For example, one of the chiefs interviewed explained that nothing he knew about vaccines would deter him from accessing the services, and a women's leader said she knew it was her responsibility to take her child to be vaccinated. Similar views were shared in FGDs with older women and younger parents, who explained that they had not experienced major problems in accessing vaccination services.

The majority of discussants held the view that the main benefit of immunization was avoidance of disease-related child death and disability and reduction in the severity of disease. Some participants said they understood that vaccines worked for a limited time, while others said that vaccines ensured that children did not get infections too frequently. Some misconceptions among parent FGD discussants included statements that vaccines can cure diseases. In addition, community health workers in one focus group in Busia reported that mothers sometimes confused vitamin A injections--given to babies during Malezi Bora (good nurturing) Week--with vaccination.

#### Motivating factors related to immunization

Many of the perceived benefits of immunization were also seen as motivating factors for getting children vaccinated. Within this context, parents pointed to their understanding that vaccines protected children from specific dreaded diseases. Parents in both the Coast and Busia regions also voiced appreciation for the health education provided by service providers about vaccination, and they welcomed the endorsement of influential community members, including chiefs and religious leaders. Some parents, particularly mothers, said that seeing a health care provider for vaccination might provide an added benefit to their children in the detection of previously undiagnosed health problems.

#### Constraints to child immunization

While parents were generally positive about Kenya's immunization programs, they also provided insights into why some children may not get vaccinated. In this regard, many pointed to a lack of understanding among some people of the benefits of vaccination:

*There are those who if they are told that there is a vaccine being given, they do not go. They say, "What is it for?" They do not understand what it is for, therefore do not let their children go for it*. (Participant, FGD fathers 50+, Kinango, Coast)

A community health worker in Busia offered another perspective, pointing to the role of mothers-in-law:

*They may argue their son (now father to her grandson) was not vaccinated and yet he is strong and healthy*. (Participant, FGD community health workers, Butula, Busia)

Among constraints to immunization, parents in FGDs in both Busia and the Coast voiced a variety of concerns about the potential side effects of vaccination and about perceived injection practices. Side effects described as common included soreness at the vaccination site and slight fevers, while serious side effects included abscesses at the vaccination site, which were attributed to poor injection technique. Parents in both Busia and the Coast said that they preferred to be served by experienced service providers as opposed to "dressing staff," and some participants said they were concerned that needles and syringes were reused on different children. In a related concern, several participants suggested that parents may worry about taking children to be vaccinated because they fear the child might become infected with HIV through unsafe injection practices. Parents in Busia and the Coast also highlighted fears that the government may be using vaccines to sterilize young female children or to reduce the population.

Certain traditional cultural practices may inhibit timely immunization, especially with regard to vaccines given soon after birth. In the Busia region, study participants reported that mothers who deliver at home are required to keep the baby indoors for three to four days after birth (three days for a girl, four days for a boy). In addition, some mothers do not like their children to be weighed naked during a check-up or to have them share the weighing basket with others. Study participants noted that some religious denominations also forbid childhood immunization.

#### Complaints about access to vaccine services were voiced by parents in both Busia and the Coast

While parents could list places where children could be vaccinated, some felt that clinic services were at times difficult to access. Some also said they did not get sufficient advance notice about vaccination days. Parents and other caregivers also complained of the inconvenience of traveling to a clinic and then waiting for hours only to be told that services were not available because of stockouts of vaccines and other drugs. For their part, many service providers interviewed said they were sometimes unwilling to open vaccine vials with short expiration dates if there were not enough children to use all the doses.

The perceived attitudes of some service providers toward patients also appeared to affect willingness to attend a clinic. Some parents were reportedly concerned that they could be criticized for not dressing their children well, for following traditional health practices, or for having babies wear items to protect them against harm. Young fathers in Busia Township explained that dirty clothes worn by a child or mother or failure to cover a baby with a shawl sometimes elicited criticism from service providers. Several mothers also feared that they would not be welcome at vaccination centers if they had not shown up for antenatal care or had not delivered their babies at a clinic.

Participants in several parent FGDs suggested that some women seemed to be the focus of negative reactions at vaccination centers for reasons that had to do with their parenting choices. Reports of some mothers being scolded by health care providers or even other women for having given birth at close intervals, for having several children to vaccinate at the same time, or for being pregnant while still breastfeeding were heard during FGDs with parents in all age groups and settings. For mothers in Samia, being pregnant past the age of 45 was said to elicit frowns and scorn at vaccination centers.

For their part, service providers spoke of feelings of frustration when parents did not come in for vaccination. Such frustration could lead to unfriendliness or scolding. Some providers and many parents described families who had not taken their children to be immunized as "lazy," "ignorant," or "difficult." A Busia health worker captured the frustrations of other health care providers:

*They are careless parents. Continued visitation or education through a close friend will help change them. Such parents do not have proper knowledge of the benefits of vaccines. Education should be done through local barazas *(formal open meetings called by local area chiefs and other administrators) *and churches for them, to help protect the life of the innocent child*. (Participant, FGD community health workers, Butula, Busia)

Parents' instinctive calculation of opportunity costs and benefits often influences whether or not they take their children to be vaccinated. A closer look at the study data indicates that parents, particularly women in the Coast region, are strongly motivated when vaccinations are free, when no payments are required at the clinic, and when incentives, such as bed nets, are provided. For some mothers in Busia, the real motivation is not that the child should be vaccinated, but rather the desire for bed nets, food, milk, or other free goods provided as incentives during vaccination sessions.

### Future malaria vaccines: issues and concerns

During FGDs, key informant interviews, and clinic exit interviews, field researchers oriented participants on progress in malaria vaccine development and asked them to respond to the prospect of a malaria vaccine. By and large, participants were excited about the potential of a malaria vaccine, with no major differences found by region, gender, or age group. At the same time, questions and concerns surfaced.

#### Reactions to the possibility of a malaria vaccine

Responses generally reflected the view that a malaria vaccine would bring added health benefits. Many participants, including district health officers, noted that communities would welcome such a vaccine if it were easily accessible. In many discussions, parents did not see a problem with a new vaccine "fitting in" with existing control measures. Parent discussants commonly held the view that reductions in the number and severity of malaria cases would indicate that a vaccine was working. These comments by a Busia father and a Coast mother sum up some of the reactions:

*It will complement the existing interventions. The vaccine, net, and cleanliness should be combined to fight malaria*. (Participant, FGD fathers 50+, Bunyala, Busia)

*There is a lot of malaria in Kinango, especially now during the rainy season.... Therefore, if that vaccine comes, all of us will feel some relief. And we are really eager for it! [Laughter] *(Participant, FGD mothers 25-49, Kinango, Coast)

#### Specific questions and concerns

Participants also expressed concerns and raised questions about a malaria vaccine. Some parents worried that introduction of a vaccine could signal that current malaria drugs were no longer effective. Others wondered whether the availability of a vaccine could reduce the need for bed nets. Some participants were more guarded in their response to the possibility of a malaria vaccine, suggesting that whether or not they accepted one would depend both on the advice they received from service providers (collectively referred to as "doctors") and on what they observed about the vaccine's effectiveness. In many discussions, parents said they would want to know where a vaccine was tested, on whom, and what had become of those on whom it was tested. Many responses suggested that vaccine developers should target everyone, including pregnant women and the elderly and that such a vaccine should be delivered orally. Commonly asked questions included how a vaccine would work in the body, when it would be introduced, and whether it would treat or prevent malaria (see Table 4).

**Table 4:** Information needs related to a future malaria vaccine.

Information requested by all participants
• Malaria prevalence rates.
• Expected benefits of the vaccine.
• Whether the vaccine would prevent or cure disease.
• Whether the vaccine would provide complete or partial protection.
• Number of doses needed.
• Mode of administration.
• Possible side effects of the vaccine.
• How the vaccine would work; how it would differ from other vaccines in use.
• Whether the vaccine would be for children only or for pregnant women and others as well.
• Ages of children to be vaccinated.
• Duration of protection and need for booster doses.
• Whether the vaccine would be offered at anytime or only during malaria outbreaks.
• Where vaccinations would take place.
• Whether people would have to pay for the vaccine.
• Whether vaccination would be accompanied by incentives (such as bed nets).
• Manufacturer of the vaccine.
• Where and when the vaccine was tested and the outcome.
• Whether the vaccine was the result of a governmental or nongovernmental initiative and which governments support it.

**Information requested by health workers only**

• How to administer the vaccine.
• Dosage.
• Vaccine storage and handling requirements.
• Expiry date.

#### Expectations for a new vaccine

In brainstorming about what they would like from a malaria vaccine, FGD participants emphasized the idea of protection, which they said should be more beneficial than the protection afforded through existing malaria interventions. Judging from the data, there was a general expectation that the protection would last at least six months; whereas for some, the vaccine should have a life-long effect. In some discussions, the view that communities would not mind the introduction of a partially efficacious vaccine was evident. One service provider noted that most vaccines did not have 100 percent efficacy. Another viewpoint held that as long as a vaccine boosted people's immunity (including children's), percentages would not matter. One provider summed up this view in the following way:

*I don't think they would mind.... [People are not highly educated and] describing the percentage is not viable, as long as the child has been immunized.... Even if the efficacy is low, I do not think there will be a problem with the uptake*. (District Medical Officer of Health, key informant interview, Coast)

Equally numerous, however, were those voices suggesting that a vaccine that is only partly efficacious could be negatively received by the community. Comments reflecting this view were observed across all study groups. The data suggest that many participants interpreted the concept of efficacy in terms of a "weak" or "strong" vaccine. For example, a health administrator in the Busia region said:

*The idea of a vaccine that is not strong or effective enough will discourage people from accessing the service for fear of being harmed by the vaccine*. (Health administrator, key informant interview, Busia)

Similarly, a father in Mombasa stated:

*If you show us the weakness of the vaccine, then we will feel cheated and ignore it*. (Participant, FGD fathers 25-49, Mombasa, Coast)

Concerns around affordability of a new vaccine surfaced in many parent discussions. Even though there is no charge for vaccines in Kenya, participants discussed affordability within the context of expenses related to clinic visits; for example, transportation to and from health facilities and unofficial charges for services at clinics. Many participants said they felt that a malaria vaccine should be free and that its adoption should help households to reduce expenses through a reduction in malaria-related illnesses. Participants noted that access to a malaria vaccine should be easy and that it should be given at the same time as other childhood immunizations. Others hoped that a malaria vaccine would be available at all health facilities and that patients would receive milk or other incentives following vaccination. Such suggestions were noted more commonly in group discussions in the Coast region.

Some participants voiced strong opinions on the need to see community health workers work hand in hand with service providers to reduce clinic workloads and wait times. Other ideas reflected the desire for broader community involvement in vaccination campaigns. For example, young parents (particularly in the Coast region) voiced a desire to be involved in campaigns for a new vaccine.

## Discussion

Findings of this study support the view long held by health economists [22] and more recently by leading social scientists that introducing a health intervention does not, on its own, guarantee acceptance by communities. In the case of a vaccine, acceptance may depend on a number of factors that, if not addressed, may result in suboptimal immunization coverage [8,10,14,23-26]. Some of these factors may constitute potential constraints to vaccination, while others could potentially enable or motivate those who influence vaccine use. Still other factors relate to gaps in information about the intervention or the disease. The findings of this study highlight the importance of understanding these kinds of sociocultural and health communications issues related to existing malaria interventions and to vaccination [10,13] in order to inform a communications strategy and policy decisions regarding vaccine introduction.

### Potential constraining factors

A clear set of issues emerged from this study as potential constraints to vaccination. Some issues relate to the overall quality of services at health facilities (congestion, delays, and the perceived attitudes of some service providers) and highlight the critical need to fast-track skills-building related to customer care, especially in light of increased community awareness of an individual's rights to health [8,13,27]. Studies have shown that sustained levels of vaccination coverage are only possible when the quality of services is perceived to be good [13,15]. In fact, several studies over the past decade have shown that persistent demand for improved vaccination delivery services exists worldwide [10,28]. Concerns about vaccine side effects within the context of concerns about the health and development of a young child also were raised by parents in this study, and have been heard among parents in other low-resource settings [10,14,15].

### Potential motivating factors

Participants strongly underscored the roles played by certain community members and health care workers as both providers of health-related information and as endorsers of vaccination services. Of particular significance is the potentially influential role of the community health worker, who is viewed as an extension of the health delivery system under Kenya's new community health strategy. In addition, District Medical Officers of Health explained that health service providers were highly regarded by the community and could be key influencers, particularly in communities with low levels of health awareness and literacy.

### Information gaps

A well-designed communications strategy would be essential to fostering a supportive environment for an eventual malaria vaccine. Effective communication would highlight the characteristics of the new intervention, address community questions and concerns prior to introduction, and build trust in vaccination [9,15,21,29-34]. The strategy would include key messages (addressing information needs as well as potential motivating and constraining factors with regard to vaccine use), and it would outline effective ways to deliver these messages through information channels to identified target audiences.

### Key messages

Results suggest that while there is much on which to build in terms of current knowledge of the malaria burden and the generally positive image of immunization, additional information needs exist:

• The finding that fever may be equated with malaria and that other illnesses with fever as a symptom may be seen as malaria should be addressed. This finding has implications for how communities may eventually judge future malaria vaccine efficacy as well as efficacy of current malaria control methods.

• Specific messaging should highlight the potential role of a vaccine within a comprehensive malaria control strategy. This should help to address concerns that malaria vaccine introduction would be perceived as obviating the need for other malaria interventions.

• Managing expectations about a new vaccine should be a key aspect of a communications strategy. Participants articulated many unrealistic expectations and assumptions about what an eventual malaria vaccine might offer.

• Specific information about a malaria vaccine should be provided. Information needs identified relate to how a new vaccine would work, its safety profile, duration of protection, when and where to access the vaccine, and target age groups.

• Key messages should aim to strengthen interactions between service providers and community members, including younger and older parents and those less economically privileged.

• Messages should consider "hidden" costs identified within communities, such as transportation charges and unofficial fees for services, as these could affect the perception of whether or not a vaccine is affordable.

### Target audiences

The results show that parents and other caregivers of children often are influenced by a broad network of people within communities in making decisions around childhood vaccination. This suggests that a communications strategy supporting vaccine introduction should target men as well as women, community and health workers, and political, civic, and religious leaders.

### Information channels

Community health workers, traditional healers, and health care providers emerged as some of the trusted sources and key influencers within the communities and should be considered as important channels for health communication. Shopkeepers and herbalists should also be considered.

### Study limitations

This study has several important caveats to be kept in mind. To meet the study aims, an exploratory, qualitative approach was employed using criterion-based sampling techniques entailing a small yet carefully selected sample. The merits of this study should, therefore, be judged according to standards appropriate for qualitative research. The findings reflect the experiences of communities located in two regions of Kenya only, the Busia and South Coast regions of Coast and Western Provinces. In either case, it is not appropriate to generalize findings to the entire Kenyan population.

## Conclusions

As Kenyan policymakers gather data for decisions on possible malaria vaccine introduction, a number of factors should be considered. First, study participants understand that malaria is a serious problem and that no single tool can control the disease. Communities therefore welcome the prospect of a vaccine against malaria, although they would have questions and concerns about the potential of the new intervention. It is also noteworthy that when deciding whether or not to have their children vaccinated, parents weigh a number of factors, including personal opportunity costs, resource constraints, and perceived benefits.

The study raises other important points that can inform both a well-grounded communications strategy and policy decisions, should a malaria vaccine become available for use. For instance, while a strong appreciation for local child immunization programs exists, participants have concerns about the quality of services offered and have ideas about how they can improve. Such views do not reflect serious dissatisfaction; rather, they represent empowered communities who worry that too many children have yet to be reached with immunization services. Finally, targeted health systems strengthening--particularly as it affects interactions between health care providers and community members--should be addressed.

## Competing interests

The authors declare that they have no competing interests.

## Authors' contributions

DIO (Principal Investigator) was responsible for overall study design, management, analysis, and writing. YWM, PO, and EY (Co-investigators) were equally responsible for the design, management, and analysis in the study. ABN, AB, YC, and PV provided technical oversight to the study, which included reviewing the protocol, data quality checks, and reviewing as well as writing parts of the manuscript. All authors read and approved the final manuscript.
